# Poly (Vinyl Butyral-Co-Vinyl Alcohol-Co-Vinyl Acetate) Coating Performance on Copper Corrosion in Saline Environment

**DOI:** 10.3390/molecules25030439

**Published:** 2020-01-21

**Authors:** Adriana Samide, Claudia Merisanu, Bogdan Tutunaru, Gabriela Eugenia Iacobescu

**Affiliations:** 1 Department of Chemistry, Faculty of Sciences, University of Craiova, 107i Calea Bucuresti, 200478 Craiova, Romania; samide_adriana@yahoo.com (A.S.); claudia.merisanu@yahoo.com (C.M.); 2 Doctoral School of Sciences, Faculty of Sciences, University of Craiova, A.I. Cuza no.13, 200585 Craiova, Romania; 3 Department of Physics, Faculty of Sciences, University of Craiova, A.I. Cuza no.13, 200585 Craiova, Romania; gabrielaiacobescu@yahoo.com

**Keywords:** adsorption, coatings, poly (vinyl butyral-co-vinyl alcohol-co-vinyl acetate), corrosion tests, atomic force microscopy

## Abstract

Poly (vinyl butyral-co-vinyl alcohol-co-vinyl acetate) named further PVBA was investigated as a protective coating for copper corrosion in 0.9% NaCl solution using electrochemical measurements such as, electrochemical impedance spectroscopy (EIS) and potentiodynamic polarization associated with atomic force microscopy (AFM). The PVBA coating on the copper surface (Cu-PVBA) was modeled in methanol containing PVBA. Its inhibitory properties against corrosion was comparatively discussed with those of the copper sample treated in methanol without polymer (Cu-Me) and of untreated sample (standard copper). A protective performance of PVBA coating of 80% was computed from electrochemical measurements, for copper corrosion in NaCl solution. Moreover, AFM images designed a specific surface morphology of coated surface with PVBA, clearly highlighting a polymer film adsorbed on the copper surface, which presents certain deterioration after corrosion, but the metal surface was not significantly affected compared to those of untreated samples or treated in methanol, in the absence of PVBA.

## 1. Introduction

Corrosion control of metals/alloys, as well as the choice of corrosion inhibitors are important issues facing certain industrial fields. Production, synthesis, and transportation of corrosive products lead to the failure of metallic materials resulting in environmental and economic problems. Certain corrosion protection strategies take into consideration assembling of some surface coatings using the inorganic and organic polymers with inhibitory properties and low costs, thus retarding the destructive effects caused by the metal oxidation processes [1,2,3].

In this regard, eco-friendly, natural, and synthetic polymers were successfully used to protect different metallic substrates against corrosion [1,2,3,4].

An effective material for corrosion protection of 2024–T3 aluminum alloy [5] was obtained by encapsulating of some organic inhibitor molecules in polylactic acid/polysiloxane hybrid nanoparticles. An epoxy resin polymer with aluminum oxide insertions having a self-regeneration ability was tested for aluminum corrosion protective coating [6].

Graphene and graphene/epoxy ester-siloxane-urea hybrid polymer nanocomposite were tested by potentiodynamic polarization and electrochemical impedance spectroscopy to determine the corrosion resistance of coated aluminum alloy [7,8]. Moreover, graphene oxide functionalized with acrylamide/acrylic acid copolymer provided a good corrosion protection for the magnesium alloy surface [9]. Furthermore, by potentiodynamic polarization and electrochemical impedance spectroscopy, the high value of coating protection efficiency of 99.8% was computed. On the other hand, biodegradable magnesium alloys have been coated with polymeric thin films (polyamide, polyacrylic acid, polylactic acid, polydopamine, chitosan, collagen, poly (lactic-co-glycolic) acid, polycaprolactone, enhancing their corrosion resistance [10,11,12].

Special attention was paid to steel corrosion processes and implicitly to its protection with polymeric films. The environmentally polymers with self-regeneration properties were studied to improve the corrosion resistance by their adsorption on the steel surface [13,14,15,16].

The copolymers represent a specific class of polymers, which also were used for coating modeling at metal–environment interface to obstruct the corrosion [17,18,19,20].

The copolymer as poly (maleic anhydride-co-*N*-vinyl-2-pyrrolidone), poly (3,4-ethylenedioxythiophene-co-indole-5-carboxylic acid), poly (methyl methacrylate-co-*N*-vinyl-2-pyrrolidone), and polyaniline/polystyrene/polybutadiene were reported as effective corrosion inhibitors [17,18,19,20]. The electrochemical measurements, UV–Vis spectroscopy, X-ray diffraction, X-ray photoelectron spectroscopy, scanning electron microscopy, Raman and Fourier-transform infrared spectroscopy, and thermogravimetric analysis were employed to investigate the composition and efficiency of polymer films adsorbed under various conditions and changing the steel surface features [21,22,23,24,25,26,27].

Copper has many applications in various fields due to its mechanical and electrical properties. Copper has a good breakage resistance and ductility being easily molded at high pressures. Moreover, it has a relatively low hardness and high thermal conductivity, being used in the manufacture of boilers and other devices involving the heat transfer, as well as of some cables, pipes, bushings, bolts, and chemical equipment. At the same time, pure copper is widely used in electrotechnics, when making coils and electromagnets [28,29,30]. Additionally, copper has antibacterial properties determining its use in the manufacture of household vessels, which have successfully replaced those of stainless steel [28]. In the presence of chloride ions, copper is susceptible to different corrosion types such as pitting and local level, which affects the metal surface characteristics, existing the risk of the release of a small amount of copper in the food affecting the human health [28]. Copper is known to be an essential element for the normal human body functionality, but ingested in excess, it can cause toxic effects [28]. Thus, it is necessary to study the copper corrosion in simulated saline environments, as well as the application of some strategies of the metal surface protection restricting the copper corrosion processes.

Polymer coatings such as polyvinyl acetate [28], polyvinyl alcohol [29], polyamide [31], and chitosan [32] provide effective protective coatings for copper corrosion in different media. Their anticorrosion performance depends on the environment composition and its pH, as well as the polymer solubility in a corrosive environment and method by which the coatings were obtained. As for example by electropolymerization, when the potential selected is very important [33], a value of around 77% [28] was obtained for inhibition efficiency of polyvinyl acetate [28] or by electrodeposition [29,30,31,32] from inhibited media when the efficiency reached different levels, ranging from 89% for chitosan [32], 92% for polyamide [31], and 94% for polyvinyl alcohol [29]. Polymer networks based on vinyl butyral units presents interesting property as the “shape memory” [34,35], having the ability to recover the initial shape upon exposure to an external thermal, chemical, magnetic, mechanical, or electrical stimulus [36]. The multifunctional shape memory polymers possess also self-regeneration, anticorrosion, drug delivery, or other properties [36,37]. The polymers based on poly (vinyl butyral) have many applications, including the manufacturing of some materials for automotive safety glasses, encapsulation of solar modules, or as a binder for different coatings, adhesives, paintings, enamels, and inks. The functional groups from macromolecular chain are responsible for its adsorption properties on various materials through covalent and/or hydrogen bonding.

In the current study, the PVBA film protective performance against copper corrosion in a saline environment was investigated by electrochemical impedance spectroscopy (EIS), potentiodynamic polarization, and atomic force microscopy (AFM). Three types of copper samples were studied, as follows: (1) Standard copper; (2) copper treated in methanol without polymer denoted as (Cu-Me); (3) copper treated in methanol containing copolymer named further PVBA (Cu-PVBA).

PVBA is a good choice, because it is a continuation of our previous studies [28,30,38] where polyvinyl acetate [28] and polyvinyl alcohol [30,38] were investigated as copper corrosion inhibitors in environments containing Cl^−^ ions. On the other hand, the studied copolymer contains the structural units of polymers above and vinyl butyral units. The latter contain oxygen active centers through which it can be bonded to the copper surface and may act by synergic adsorption mechanism with the other structural units from the macromolecular chain.

PVBA film deposition was performed from a methanolic solution due to the good solubility of the copolymer in this solvent. In order to determine the methanol effect on the copper surface, as well as its influence on copper electrochemical behavior, two control samples were used, namely the untreated standard copper (Cu) and the copper treated in methanol (Cu-Me).

## 2. Results and Discussion

### 2.1. Open Circuit Potential Discussion

**Figure 1:**
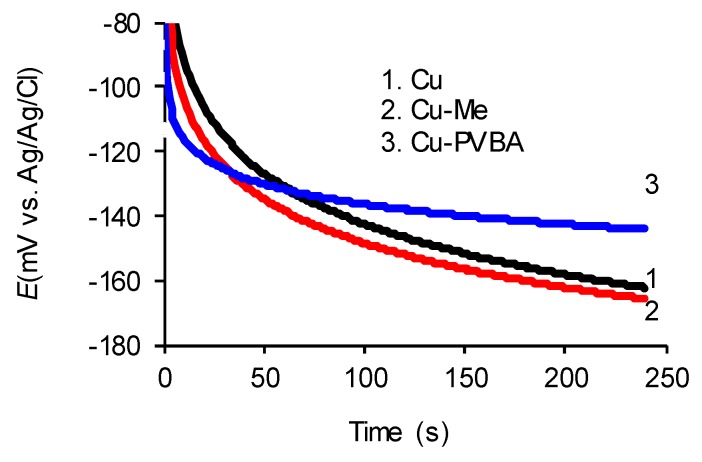
Variation of the open-circuit potential for standard copper, methanol-treated copper, and poly (vinyl butyral-co-vinyl alcohol-co-vinyl acetate) (PVBA) modified copper, recorded in 0.9% NaCl solution.

The potential variation at open circuit was recorded, as shown in Figure 1. A similar behavior is observed of the Cu-Me sample with the standard one (curves 1 and 2), meaning that a slowly potential decrease over time takes place, with a stabilization tendency in respect with an exponential function.

In contrast, in the presence of PVBA (Cu-PVBA sample) in NaCl solution, the open circuit potential stabilizes after a few minutes around the value of −140 mV. This is higher by about 20 mV than those of the standard copper and Cu-Me samples, indicating that the surface characteristics were changed due to PVBA adsorbed on the surface leading to the restriction of the ion exchange at the metal-electrolyte interface. Thus, there is a relative surface passivation due to the polymeric film leading to the OCP displacement at higher value than those of control samples. Keeping the OCP at the same value (around −140 mV) suggests that the copolymer desorption did not occur, PVBA being anchored by the substrate, thus retarding the formation of corrosion products on the copper surface.

In the absence of PVBA film the shape of curves may indicate that the balance at copper/electrolyte interface establishes harder because of the formation of an unstable surface layer. Moreover, the copper cations formed at the metal/solution interface follow a surface adsorption–desorption cycle.

### 2.2. Electrochemical Impedance Spectroscopy (EIS)

**Figure 2:**
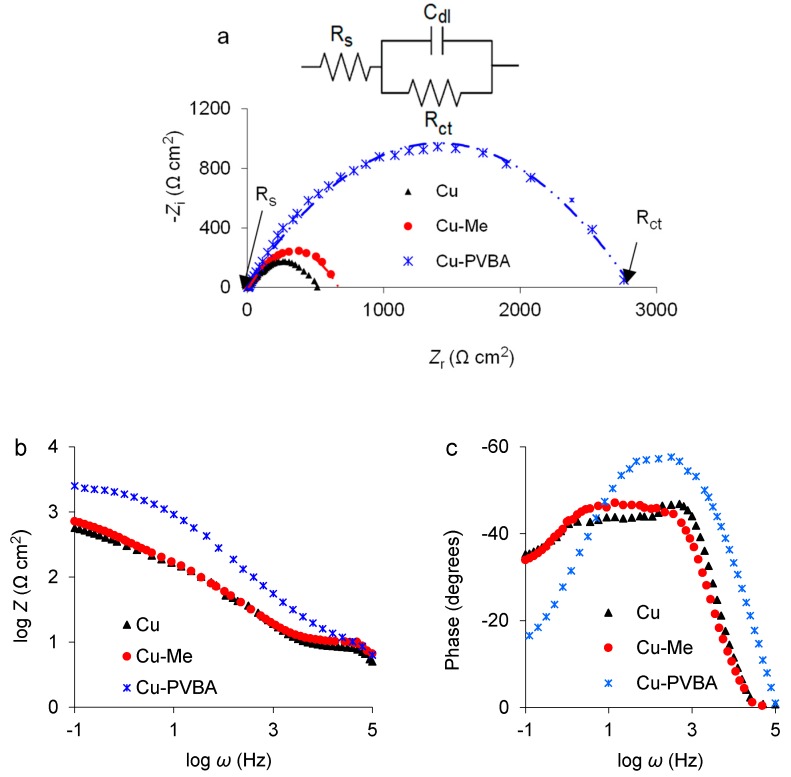
Nyquist (**a**), impedance Bode (**b**), and phase Bode (**c**) diagrams for standard copper, methanol-treated copper (Cu-Me), and PVBA modified copper (Cu-PVBA) recorded in 0.9% NaCl solution.

As shown in Figure 2, classical Nyquist diagrams (Figure 2a) were obtained for all three samples immersed in sodium chloride solution, highlighting capacitive loops with approximately semicircular shapes whose diameter gradually increases, reaching the highest value for Cu-PVBA sample. The capacitive loop intersection with the impedance real axis (Z_r_) at very low frequency (0.1 Hz), represents the charge transfer resistance (R_ct_) while the intersection with the real axis at very high frequency (100 kHz) represents the solution resistance (R_s_) [39], as shown in Figure 2a.

The Nyquist impedance curves indicate that the copolymer macromolecules were adsorbed on the metal surface, modeling a surface film that leads to the increase of copper impedance response (Z_r_) and by default the charge transfer resistance (R_ct_) [28,40,41].

The Bode diagram (Figure 2b) designs for impedance, almost overlapped curves for standard copper and the one treated in methanol and a completely distinct one for Cu-PVBA, having the highest response at the lowest frequency value of 0.1 Hz (logZ = −1), being in good agreement with Nyquist plots.

On the other hand, the phase Bode diagram (Figure 2c) shows that: (i) Standard copper phase shows a maximum at 46.3 degrees centered around a well-defined frequency of 631 Hz (logZ = 2.8), then it forms a plateau corresponding at about 44.5°, in the frequency range between 1 and 100 Hz (logZ ranging between 0 and 2).

Moreover, the phase decreases in the lower frequency region with respect to the plateau value. (ii) Phase in the Cu-Me case is very similar to the Cu standard case, with only two main differences, i.e., no peaks are present and the plateau value, in the frequency range from 1 to 100 Hz about 48.8°, is a little bit higher, but it is almost identical in the low frequency region. The slightly different behavior from that of the standard copper is probably due to the chemically finished metal surface in methanol. (iii) In the Cu-PVBA case, phase shows a well-defined maximum (57.1°) in the 100–1000 Hz range corresponding to the logZ values between 2 and 3 and strongly decreases at lower frequencies, from 0.1 to 10 Hz, i.e., for log Z values varying from −1 to 1. Moreover, in the presence of PVBA, the phase drops to zero at far higher frequencies (100 kHz, logZ = 5) than in the standard and the Cu-Me case (31.6 KHz, logZ = 4.5).

Those mentioned above indicate a different chemical composition of the upper layer [42] due to the PVBA macromolecules adsorbed on the surface, which drastically changes the metal behavior to corrosion.

The determination of the electrochemical parameters from EIS was performed by fitting the experimental data according to a simple circuit (inserted in Figure 2a) consisting of three elements, as: Charge transfer resistance (R_ct_) connected in a parallel position with the double-layer capacitance (C_dl_), both linked in series with the solution resistance (R_s_). The coating protection performance (P%) of PVBA was computed from both Equations (1) and (2) [28]. The results are listed in Table 1.

(1) $$P\%\;=\;\left(1\;-\;\frac{Z^{S}}{Z_{\mathrm{PVBA}}}\right)\;\times \;100$$

where Z^s^ is the impedance response in the PVBA coating absence and Z_PVBA_ represents the impedance response in the presence of PVBA adsorbed coating on the copper surface.

(2) $$P\%\;=\;\frac{R_{\mathrm{ct}}^{\mathrm{PVBA}}{\;-\;R}_{\mathrm{ct}}^{S}}{R_{\mathrm{ct}}^{\mathrm{PVBA}}}\;\times \;100$$

where $R_{\mathrm{ct}}^{S}$ is the charge transfer resistance of standard sample and $R_{\mathrm{ct}}^{\mathrm{PVBA}}$ represents the charge transfer resistance of Cu-PVBA sample.

Analyzing the data from Table 1, it can be observed that there is a good correlation between the impedance responses and the charge transfer resistance, their highest values being obtained in the case of Cu-PVBA sample, when the lowest values were recorded for C_dl_ and R_s_.

PVBA film protection performance reached a value of about 81.1% calculated from both Equations (1) and (2), mentioned above.

### 2.3. Potentiodynamic Polarization

**Figure 3:**
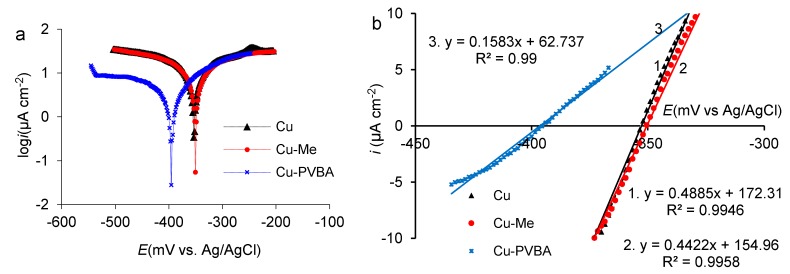
Potentiodynamic polarization curves recorded with a potential scan rate of 1.0 mV s^−1^, on standard copper, methanol-treated copper, and PVBA modified copper, in 0.9% NaCl solution, at room temperature: (**a**) Semilogarithmic curves; (**b**) linear diagram drawn in the potential range close to corrosion potential (± 20 mV).

After EIS, the potentiodynamic polarization was applied on the three electrodes namely standard copper (Cu), methanol treated copper (Cu-Me), and PVBA modified copper (Cu-PVBA) registering both diagrams (Figure 3), the semilogarithmic (Figure 3a) and the linear one (Figure 3b).

As shown in Figure 3a, the Cu-Me sample similarly behaves to the standard one. The potentiodynamic curves are framed within the same potential range and current area, which led to their apparent overlap, with a slight displacement of the one corresponding to Cu-Me towards lower current density. The presence of PVBA molecules adsorbed on the copper surface leads to the shifting of potentiodynamic curves to negative direction and lower current density compared to those discussed above. Consequently, the polymer coating adsorbed on the copper surface leads to the corrosion current density decrease and polarization resistance increase as it is demonstrated from linear diagram (Figure 3b) recorded in the potential range (± 20 mV) close to corrosion potential (E_corr_). The corrosion current density (i_corr_) was computed at Tafel lines intersection extrapolated to corrosion potential, the best fitting of potentiodynamic data being in the potential range of ± 250 mV, in respect with E_corr_. The anodic and cathodic Tafel slopes (b_a_ and b_c_) were determined with a mean squared deviation, R^2^ of 0.99.

By deriving the equations inserted in Figure 3b, the slope of the straight lines drawn as tangents to the polarization curves recorded in a potential range close to the corrosion potential was computed [41,43]. Thus, the polarization conductance (S_p_) was determined from the corresponding line slope (di/dE) [41,43] and implicitly the polarization resistance (R_p_) was deduced as 1/S_p_ [41,43]. The electrochemical parameters are shown in Table 2.

Analyzing the values of the electrochemical parameters, it was found that the methyl alcohol does not have a protective effect on the copper surface, but rather represents a degreasing agent and consequently, the corrosion current computed for Cu-Me maintained at approximately the same value as in the standard case. The polarization resistance of the Cu-PVBA sample increases significantly compared to both standard and Cu-Me samples, indicating that the polymer layer is stable and adherent on the copper surface. The Tafel anodic slope (b_a_) has the smallest value for Cu-PVBA sample, showing that protection takes place by blocking the surface-active sites via copolymer adsorption. In case of the Cu-Me sample, the metal dissolution occurs with the anodic larger Tafel slope revealed by the highest value of corrosion current density. The values of the Tafel cathodic slopes (b_c_) indicate that the treated surfaces influence the cathodic reaction evolution.

The layer protection performance (P%) calculated with Equation (3), has maintained at a high level, very close to the one calculated from the EIS, meaning that during the impedance measurements, very limited desorption process of PVBA took place and consequently, the polymer molecules are strongly linked from copper substrate.

(3) $$P\%\;=\;\frac{i_{\mathrm{corr}}^{S}{\;-\;i}_{\mathrm{corr}}^{\mathrm{PVBA}}}{i_{\mathrm{corr}}^{S}}\;\times \;100$$

where $i_{\mathrm{corr}}^{S}$ is the corrosion current density of standard sample and $i_{\mathrm{corr}}^{\mathrm{PVBA}}$ represents the corrosion current density of Cu-PVBA sample, respectively.

Thus, it can be certainly stated that the PVBA molecules are strongly adsorbed on the substrate through numerous adsorption centers existing along the macromolecular chain such as oxygen atoms.

### 2.4. PVBA Adsorption Mechanism

**Figure 4:**
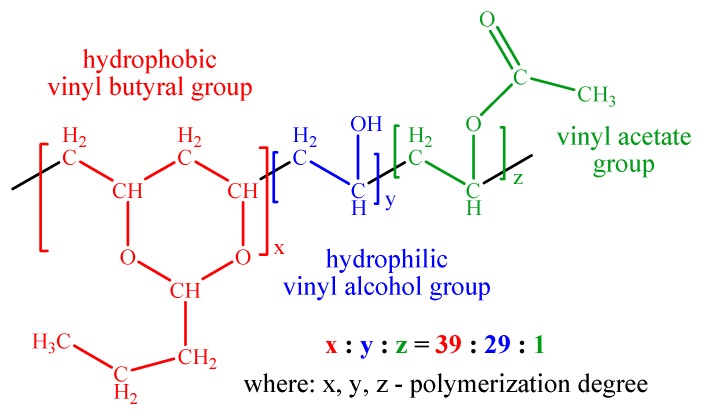
Molecular structure of poly (vinyl butyral-co-vinyl alcohol-co-vinyl acetate).

Analyzing the PVBA molecular formula (Figure 4), three distinct structural units are observed, as follows [35]: The hydrophobic groups (in a highest proportion) corresponding to the polyvinyl butyral macromolecular chain; hydrophilic groups from polyvinyl alcohol; in a smaller proportion, the acetate groups from polyvinyl acetate.

In context with the polar and nonpolar character of the mentioned groups, PVBA has adhesive properties with different materials such as glass, metals, and wood [35] through “hydrogen bonds (noncovalent interactions), metal coordination, host-guest interactions, ionic attractions, hydrophobic interactions” as shown by Zhi-Chao Jiang et al. in a previous study [36]. The hydroxyl groups enable the PVBA outstanding adhesion to many substrates including the metal surfaces (aluminum, brass, tin, lead, iron) increasing moisture resistance [44]. The PVBA good binding capacity on copper surface and resistance to aqueous NaCl solution are proved by the open circuit measurements (Figure 1) which show that the Cu-PVBA sample potential was stabilized to higher value compared to those of standard and Cu-Me samples, when the open circuit potential stabilization is relative when being observed at a slightly descending trend. Copper has a good ability for methanol adsorption [45] but drying of samples for a longer time favors desorption of the molecules, which leads to an electrochemical behavior close to the one of standard (Figure 2 and Figure 3).

When a perturbation appears on the Cu-PVBA sample, such as frequency variation during impedance spectroscopy, the PVBA coating ensures a significant copper surface protection in sodium chloride solution, probably due to the polymer ability to return to its predetermined shape from a temporary one, in response to the external stimulus, providing the characteristics of a shape memory polymer, as PVBA was described [34].

Consequently, by the simple dipping method of the copper sample in methanol containing PVBA the adsorption process involves two stages: (1) Initially, the adsorption of the methyl alcohol molecules on the copper surface takes place, prevailing on that of macromolecules due to steric arrangement of the polymeric chain, imposing a more restricted diffusion towards the interface; consequently, noncovalent interactions as hydrogen-bridged between hydroxyl groups from adsorbed methanol and hydroxyl groups from polyvinyl macromolecular chain can occur; (2) the hydrophobic interactions due to vinyl butyral groups represents the most likely adsorption process of PVBA macromolecules on the copper surface supplemented by a host-guest adsorption in which the copper metal network constitutes the matrix incorporating the polymer.

After the potentiodynamic polarization, the PVBA layer protection performance maintained at a similar value to that calculated from the EIS. Thus, the layer stability is preserved, the desorption of the polymer on the copper surface does not occur to an extent that affects the PVBA protective performance.

In this regard, some additional explanations are necessary. During potentiodynamic polarization, copper oxidation processes take place on the polymer-free areas. The copper ions favor the polyvinyl alcohol crosslinking reaction [29,38] and formation of some copper (I and II) complexes [29,38] which coordinatively binds on the metal surface, leading to the change of its characteristics and morphology, without affecting the polymer coating protective performance.

### 2.5. Atomic Force Microscopy

**Figure 5:**
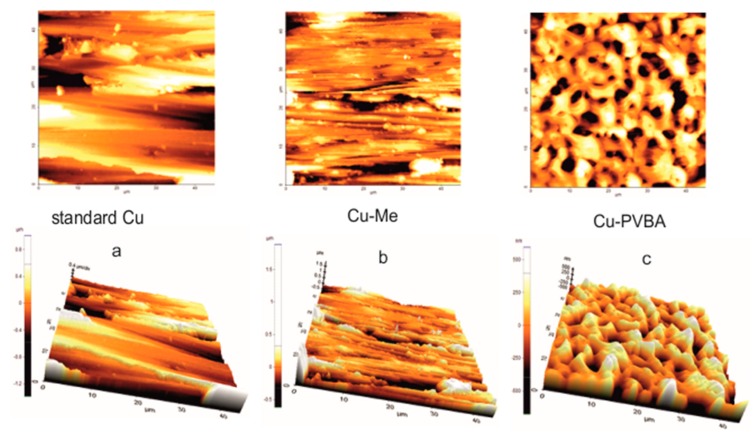
Atomic Force Microscopy (AFM) two-dimensional (2D) and three-dimensional (3D) images obtained for copper surface before corrosion: (**a**) Standard copper; (**b**) copper immersed in methanol (Cu-Me sample); (**c)** copper immersed in methanol containing PVBA (Cu-PVBA sample).

AFM 2D and 3D images were acquired before the electrochemical measurements and after potentiodynamic polarization, in order to observe the morphological characteristics of the copper surface coated with PVBA compared to those of the standard (Cu) and copper immersed in methanol (Cu-Me). Figure 5 shows the AFM images obtained before the electrochemical measurements.

Thus, three different morphological characteristics of the copper surface can be observed, namely: (i) For standard copper the AFM 2D and 3D images display a surface morphology corresponding to a mechanically processed surface (Figure 5a); (ii) the plate immersed in methanol (Cu-Me sample) preserves quite well the characteristics of the standard, however, highlighting a more even surface due to improving of its finishing. (Figure 5b); (iii) the polymer film adsorbed on the copper surface is well nuanced in Figure 5c which show a morphology completely distinct from those discussed previously, being determined by the change in both characteristics and surface chemistry.

The relatively coherent network appearance organized on the surface clearly differentiates the Cu-PVBA sample from those of the standard or treated in methanol, which demonstrates the adsorption of the polymer on the copper surface. There is virtually no similarity to the standard or the Cu-Me sample.

**Figure 6:**
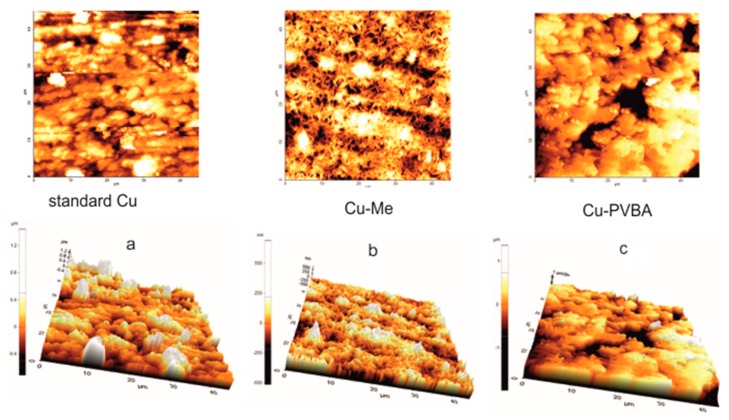
Atomic Force Microscopy (AFM) 2D and 3D images obtained for copper surface after corrosion: (**a**) Standard copper; (**b**) copper immersed in methanol (Cu-Me sample); (**c**) copper immersed in methanol containing PVBA (Cu-PVBA sample).

Figure 6 shows the AFM 2D and 3D images after the potentiodynamic polarization of the samples in 0.9% NaCl solution. In all cases, the surface appearance has changed compared to that before corrosion.

Figure 6a shows that the standard surface has deteriorated as a result of the saline solution corrosive attack. In the case of the sample treated with methanol (Figure 6b), deposits of smaller sizes occur on the surface compared to those formed on the standard (Figure 6b), probably due to a better finishing and degreasing of the surface treated with methanol inhibiting the agglomeration of the corrosion products and reducing the risk of the appearance of large aggregates. Surface morphology from Figure 6c shows that the polymer film did not desorb on the copper surface. The main difference from the one shown in Figure 5c (before corrosion) probably occurs due to the swelling of the absorbed polymer leading to apparently thicker film development. The change in coating texture can be caused by the adsorption of the crosslinked polymer and the copper-PVBA complexes which were formed during the potentiodynamic polarization, as mentioned in the previous paragraph.

## 3. Materials and Methods

### 3.1. Materials

The copper plates with the area of 2 cm^2^ (dimensions: 1 × 2 cm) were cut from a copper foil (99.9% purity) purchased from Sigma Aldrich (Steinheim, Germany). Other reagents as methyl alcohol, natrium chloride, and poly (vinyl butyral-co-vinyl alcohol-co-vinyl acetate) further named as PVBA were also obtained from Sigma-Aldrich. The PVBA composition (wt%) consists of acetate/hydroxyl/vinyl butyral, in the ratio of 1/11/88, its average molecular mass located between 90,000 and 120,000.

#### PVBA Coating Modeling

Standard copper plates were polished with sandpaper of different sizes, ultrasonically cleaned, degreased with acetone, and dried in warm air in the oven with internal air circulation, at the temperature of 60 °C (methanol boiling point—64.7 °C). After processing, the samples were immersed in methanol without and with PVBA in concentration of 6%, for 24 h, at room temperature. The samples were removed from the methanol baths and dried, for 24 h, at room temperature and 2 h in warm air.

Copper samples immersed in methanol (methanol treated copper) will be referred to as Cu-Me, while the copper samples dipped in methanol containing PVBA (PVBA modified copper) will be further denoted to as Cu-PVBA. Thus, three classes of copper samples were prepared to be submitted at corrosion in 0.9% NaCl solution and to be comparatively discussed namely, standard copper (Cu), methanol-treated copper (Cu-Me), and PVBA modified copper (Cu-PVBA).

### 3.2. Corrosion Tests

The corrosion behavior of the mentioned-above copper samples was investigated using electrochemical measurements as the open circuit potential (OCP) variation over time, electrochemical impedance spectroscopy (EIS), and potentiodynamic polarization. Atomic force microscopy (AFM) was performed to examine the changes in surface morphology.

#### 3.2.1. Electrochemical Measurements

Electrochemical impedance spectroscopy (EIS) was carried out in 0.9% NaCl solution, at a sample corresponding OCP, in the frequency range of 10^5^ and 10^−1^ Hz, with a sinusoidal perturbation, AC signal of 10 mV.

Potentiodynamic polarization was performed in 0.9% NaCl solution, at room temperature, in the potential range between −1000 and 1000 mV, with potential scan rate of 1 mV s^−1^. The standard electrochemical cell with three electrodes of 100 mL volume was used. The working electrode manufactured from copper, with an active area of 1 cm^2^, a platinum auxiliary electrode and Ag/AgCl reference electrode were coupled to an electrochemical system type VoltaLab with the VoltaMaster 4 software. The polarization curves were processed as Tafel diagram from which the corrosion current density (i_corr_) were computed at the intersection of Tafel lines extrapolated to corrosion potential (E_corr_). Moreover, the linear diagram was recorded, in the potential range close to corrosion potential (± 20 mV) and the polarization resistance (R_p_) was calculated. The experimental method and equipment were reported in our previous studies [28,29,30,38,39,40,41,42,43].

#### 3.2.2. Atomic Force Microscopy (AFM)

Atomic force microscopy was performed as in our previous studies [29,41,43], using a noncontact mode atomic force microscopy (NC-AFM, Park Systems, Suwon, Korea), PARK XE-100 SPM system). The cantilever had a nominal length of 125 mm, a nominal force constant of 40 N m^−1^, and oscillation frequencies in the range of 275–373 kHz. We used horizontal line by line flattening as the planarization method.

The AFM 2D and 3D images were obtained for: (i) Standard copper surface; (ii) the copper plates immersed in methanol (Cu-Me sample), before and after corrosion; (iii) the copper samples immersed in methanol containing PVBA (Cu-PVBA sample), before and after corrosion.

## 4. Conclusions

PVBA was chemically deposited on the copper surface using the simple dipping method of metal sample in methanol containing dissolved polymer in concentration of 6%.

The protective performance of PVBA coating on copper corrosion in 0.9% NaCl solution was investigated by electrochemical measurements such as, the open circuit potential variation, electrochemical impedance spectroscopy (EIS), and potentiodynamic polarization. The surface morphology was examined on atomic force microscopy (AFM) slides acquired before and after corrosion. The PVBA adsorption mechanism on the copper surface was also proposed.

The PVBA protective performance reached a value about of 81% computed from both EIS and potentiodynamic polarization.

PVBA acted by adsorption on copper surface involving noncovalent interactions between methanol hydroxyl groups and polyvinyl alcohol hydroxyl groups and hydrophobic interactions due to vinyl butyral groups, the metallic network being a good matrix incorporating polymer macromolecules. Moreover, during potentiodynamic polarization, crosslinked polymer and copper-PVBA complexes can occur, having a good adsorption ability on the metal substrate.

AFM reproduced before and after corrosion have a different morphology of the filmed surface compared to those of the standard and treated copper in methanol, indicating that the PVBA coating is stable and good preserved after corrosion.

## Figures and Tables

**Table 1:** Electrochemical parameters calculated from electrochemical impedance spectroscopy recorded at room temperature in 0.9% NaCl solution for standard copper, methanol-treated copper, and PVBA modified copper.

Sample	E_OCP_ (mV vs. Ag/AgCl)	R_s_ (Ω cm^2^)	R_ct_ (Ω cm^2^)	C_dl_ (µF cm^−2^)	logZ (Ω cm^2^)	Z (Ω cm^2^)	│Phase│ (degree)	P (%)
Standard	−164	245	515	845	2.71	513	46.3/peak 44.5/plateau	-
Cu-Me	−167	132	650	612	2.84	692	48.8/plateau	-
Cu-PVBA	−147	64	2763	342	3.43	2692	57.1/peak	80.9

**Table 2:** The electrochemical parameters calculated from potentiodynamic measurements for standard Cu, methanol-treated Cu, and PVBA modified Cu, in 0.9% NaCl solution, at room temperature.

Sample	E_corr_ (mV vs. Ag/AgCl)	i_corr_ (µA cm^−2^)	b_a_ (mV dec^−1^)	b_c_ (mV dec^−1^)	S_p_ 10^3^ (S cm^−2^)	R_p_ (Ω cm^2^)	P (%)
Standard	−353	10.2	179	−172	0.4885	2047	-
Cu-Me	−350	11.3	228	−232	0.4422	2261	-
Cu-PVBA	−395	2.03	133	−260	0.1583	6317	80.1

## Abbreviations

PVBA	poly (vinyl butyral-co-vinyl alcohol-co-vinyl acetate)
Cu-Me	copper treated in methanol in the absence of PVBA
Cu-PVBA	copper treated in methanol in the presence of PVBA
EIS	electrochemical impedance spectroscopy
AFM	atomic force microscopy
OCP	open circuit potential
i_corr_	corrosion current density
E_corr_	corrosion potential
R_p_	polarization resistance
R_ct_	charge transfer resistance
C_dl_	double-layer capacitance
R_s_	solution resistance
P%	coating protection performance
S_p_	polarization conductance
